# Role of matrix metalloproteinases 2 and 9, toll-like receptor 4 and platelet-leukocyte aggregate formation in sepsis-associated thrombocytopenia

**DOI:** 10.1371/journal.pone.0196478

**Published:** 2018-05-07

**Authors:** Caroline M. Larkin, Nadhim Kamil Hante, Eamon P. Breen, Krzysztof A. Tomaszewski, Simon Eisele, Marek W. Radomski, Thomas A. Ryan, Maria-Jose Santos-Martinez

**Affiliations:** 1 Department of Anaesthesia and Intensive Care Medicine, St James’s Hospital, Dublin, Ireland; 2 School of Pharmacy and Pharmaceutical Sciences and Trinity Biomedical Sciences Institute, Trinity College Dublin, Dublin, Ireland; 3 College of Pharmacy, University of Kufa, Najaf, Iraq; 4 Institute of Molecular Medicine, Trinity College Dublin, Dublin, Ireland; 5 Department of Anatomy, Jagiellonian University Medical College, Krakow, Poland; 6 Department of Pharmacy, Ludwig Maximilian University, Munich, Germany; 7 School of Medicine, University of Saskatchewan, Saskatoon, Canada; 8 School of Medicine, Trinity College Dublin, Dublin, Ireland; University of Portsmouth, UNITED KINGDOM

## Abstract

**Background:**

The development of thrombocytopenia in sepsis is a poor prognostic indicator associated with a significantly increased mortality risk. Mechanisms underlying this phenomenon remain to be clearly elucidated. Matrix metalloproteinases (MMPs) are enzymes that regulate the turnover of the extra-cellular matrix. MMP-2 is recognised as a platelet agonist with MMP-9 proposed as an inhibitor of platelet activation. The existence of MMP-9 in platelets is a subject of debate. There is limited evidence thus far to suggest that toll-like receptor 4 (TLR-4) and platelet-leukocyte aggregate (PLA) formation may be implicated in the development of sepsis-associated thrombocytopenia.

**Objectives:**

To investigate whether MMP -2/-9, toll-like receptor 4 (TLR-4) or platelet-leukocyte aggregate (PLA) formation are implicated in a decline in platelet numbers during septic shock.

**Methods:**

This was an observational study which recruited healthy controls, non-thrombocytopenic septic donors and thrombocytopenic septic donors. MMP-2, MMP-9 and TLR-4 platelet surface expression as well as PLA formation was examined using flow cytometry. In addition MMP-2 and MMP-9 were examined by gelatin zymography and enzyme-linked immunosorbent assay (ELISA) using a 3 compartment model (plasma, intraplatelet and platelet membrane).

**Results:**

There was no difference found in MMP-2, MMP-9 or TLR-4 levels between non-thrombocytopenic and thrombocytopenic septic donors. PLA formation was increased in thrombocytopenic patients. MMP-9 was detected in platelets using flow cytometry, gelatin zymography and ELISA techniques.

**Conclusions:**

Platelet consumption into PLAs may account for the development of thrombocytopenia in septic shock. MMP-9 is found in platelets and it is upregulated during septic shock.

## Introduction

Severe sepsis is often associated with a decrease in platelet numbers. With platelet counts below 150,000/μl widely accepted as thrombocytopenia [1], reported rates of sepsis-associated thrombocytopenia in the literature range from 14.5% to 70.6% [2, 3]. If thrombocytopenia does occur during sepsis it is associated with a significant increase in mortality [4–8]. Thrombocytopenia may occur due to a decreased production of platelets [9], so called “marrow failure” [10], alternatively, thrombopoiesis may be increased during sepsis [11, 12].

Matrix metalloproteinases (MMPs) are a group of zinc-dependent endopeptidases that remodel the extracellular matrix [13]. However, MMP-2 is also recognised as a mediator of platelet activation [14, 15]. Interestingly, another gelatinase, MMP-9, has been proposed as an inhibitor of platelet aggregation [16]; however, whether MMP-9 can actually be found in platelets remains a matter of debate [17–19].

Toll-like receptors (TLRs) are key players in innate immunity, acting as pathogen recognition receptors [20]. Animal studies suggest that platelet TLR-4 is involved in the development of sepsis-associated thrombocytopenia [21–23].

Following platelet adhesion to the subendothelium, adenosine diphosphate (ADP) is released from dense granules in addition to fibrinogen and P-selectin being released from α-granules. Thus P-selectin is widely regarded as a marker of platelet activation. Activated platelets can adhere to circulating leukocytes to form platelet-leukocyte aggregates (PLAs) and those are considered to be reliable indicators of a prothrombotic state. PLAs are associated with several cardiovascular disorders [24] and are also increased during sepsis [25, 26].

The main objective of this work was to study mechanisms that could contribute to the development of sepsis-associated thrombocytopenia focusing on the MMP-2/-9 platelet dependent pathway, platelet TLR-4 signalling and PLA formation in sepsis.

## Materials and methods

### Participants

Ethical approval was obtained from the Research Ethics Committee of St James’s hospital (REC reference: 2013/07/03 RTC). Consecutive patients with septic shock (as defined by the Surviving Sepsis guidelines 2012 [27]) were recruited from the ICU of St James’s hospital over an 18 month period. Exclusion criteria are listed in Table 1. Sampling took place within 72 hours of admission to the ICU. Patients’ clinical data was recorded including age, gender, diagnosis, platelet and white cell counts, microbiological culture results, Sequential Organ Failure Assessment (SOFA) score and Acute Physiology and Chronic Health Evaluation II (APACHE II) score. For classification of a patient as “thrombocytopenic” the patient had either (a) a platelet count on admission or within 72 hours of admission of less than 100,000/μl or (b) a drop in platelet count of greater than 50% from their platelet count on admission within 72 hours of that admission. We chose a limit of 100,000/μl as opposed to 150,000 because we wanted to capture clinically relevant data. A drop of >50% in platelet count is also a safe margin of error for this given that other studies have used a lesser proportional decrease. Male and female healthy controls, aged over 18, who refrained from drinking alcohol and smoking tobacco for 48 hours prior to sampling and had not taken any drugs in the preceding 14 days were recruited as controls.

**Table 1:** Exclusion criteria for patient selection.

Age below 18
Use of drugs known to affect platelet function
Cardiopulmonary bypass or extracorporeal life support in preceding 2 weeks
History of haematological malignancy
Disorders affecting thrombopoiesis e.g. essential thrombocytosis
Thrombotic microangiopathies e.g. thrombotic thrombocytopenic pupura
Pregnancy or the postpartum period
Therapeutic anticoagulation with unfractionated heparin and activated partial thromboplastin time > 40 seconds
Congenital platelet disorder e.g Bernard-Soulier syndrome
Massive transfusion of blood products (greater than their normal circulating blood volume) in preceding week
Platelet transfusion in preceding week

### Ethics statement

Ethical approval was obtained from the Research Ethics Committee of St James’s hospital, Dublin, Ireland (REC reference: 2013/07/03 RTC). Written informed consent was obtained from patients or their next of kin.

### Reagents

All reagents were purchased from Sigma-Aldrich (Dublin, Ireland) unless otherwise indicated.

### Sample preparation

Peripheral blood samples were obtained from healthy controls via venepuncture into antecubital fossa veins using 21G needles mounted on a 50 mL syringe. Blood samples from septic patients were withdrawn from indwelling central venous catheters. Whole blood was collected and processed immediately. Firstly it was mixed with 3.15% sodium citrate solution in a 9:1 ratio. Platelet-rich plasma (PRP) was prepared by centrifugation as previously described [28]. PRP was then centrifuged at 900g for 10 minutes at room temperature for the preparation of platelet-poor plasma (PPP) and platelet pellets [28]. PPP and platelet pellets were stored at -20°C and thawed for batch analysis.

### Platelet surface expression of P-selectin, MMP-2, MPP-9 and TLR-4

Flow cytometry (FC) is a laser-based technique that measures optical and fluorescence characteristics of single cells or particles. It can be used to measure the platelet surface expression of P-selectin, MMP-2, MMP-9 and TLR-4. Whole blood samples were analysed by FC within 30 minutes of collection. Primary antibodies used were CD62P APC (BD Pharmingen) (used to stain for P-selectin expression), MMP-2 PE (R&D Systems), MMP-9 (Pierce antibodies) and TLR-4 Biotin (BD Pharmingen) for platelets and CD45 PerCP (ImmunoTools) as a leukocyte marker. For TLR-4 and MMP-9, secondary antibodies anti-biotin (Miltenyi Biotec) and anti-IgG2a (Miltenyi Biotec) were used respectively. Thrombin receptor activating peptide (TRAP-6) was used as a platelet agonist. As per manufacturers’ instructions primary antibodies were incubated with whole blood in the dark for 30 minutes and secondary antibodies for 20 minutes at 4°C. Following incubation, red cell lysis was performed using an ammonium chloride/potassium bicarbonate buffer (155 mM NH_4_Cl, 10 mM KHCO_3_). Isotype controls were used to set the negative populations and experiments were run on a CyanADP (Beckman Coulter) flow cytometer. The platelet population was identified and gated based on its characteristic forward and side scatter light profile [29]. A minimum of 30,000 events were recorded per sample. Analysis of data was performed using FlowJo v10 software (Treestar) and antibody binding was expressed as the percentage of platelets positive for the antibody.

### Enzymatic activity of MMP-2 and MMP-9

Zymography is a technique for measuring enzymatic activity that is based on the degradation of their respective substrates [30], thus gelatin zymography measures the activity of the gelatinases MMP-2 and MMP-9. MMP-2 and MMP-9 gelatinolytic activity was analysed as a 3 compartment model–plasma, intraplatelet (platelet lysate) and platelet membrane (platelet homogenate). To prepare platelet lysates 0.2% (w/v) sodium chloride solution at 4°C was added to each platelet pellet, gently resuspended and kept on ice for 10 minutes. For homogenisation Triton 0.1% solution in solubilising buffer was added to each sample. Samples were centrifuged at 4°C for 10 minutes at 13,000rpm after each step and the supernatant (lysate and homogenate) kept at -80C until further used. Protein concentrations were quantified by the Bradford method [31] using the Bio-Rad Protein Assay (Bio-Rad, Alpha Technologies, Ireland). Zymography was performed as described previously [32, 33]. For plasma and lysate samples, 100μg or 30μg of protein per lane respectively, were subjected to 8% sodium dodecylsulfate-polyacrylamide gel electrophoresis (SDS-PAGE) with co-polymerized gelatin. For homogenate samples 7.9μg of protein per lane was used. Following electrophoresis, gels were washed 3 times in 2.5% Triton X-100, twice in developing buffer (50mM Tris-HCl pH 7.6, 150mM NaCl, 5mM CaCl_2_ and 0.05% NaN_3_) and incubated overnight at 37°C. Gels were stained in 40% methanol, 10% acetic acid and 0.1% (w/v) Coomassie Blue R-250 and destained in a 4% methanol and 8% acetic acid solution. The resulting gels had “clear bands” corresponding to the gelatinolytic activity of the MMPs against a blue background. The gels were imaged and quantified by scanning densitometry using a gel-documentation system (Gel Doc ™ XR+ system) and Chemidoc software (Biorad, Alpha Technologies, Ireland). The conditioned medium of HT1080 human fibrosarcoma cells was used as MMP-2/MMP-9 standards [16]. Pro- and active- MMP-2/-9 bands were measured together in units of intensity x millimetres.

### Imaging of platelet-leukocyte aggregates

Immunohistochemistry (IHC) is an imaging technique based on the binding of antigens to specific antibodies in biological specimens which was used here to image PLAs. Samples of PRP were fixed with a 10% solution of formaldehyde (pH 7.4). Samples were centrifuged and the resulting pellet dehydrated in ethanol and exchanged with xylene. Subsequently, samples were embedded in paraffin and dissected into 4μm sections. For immunohistochemical localization, formalin-fixed tissue sections were treated with 3% hydrogen peroxide. Heat-induced epitope retrieval was performed in sodium citrate buffer solution (10 mM sodium citrate, 0.05% Tween 20, pH 6.0) at 98°C for 30 minutes. Sections were incubated at room temperature with 10 μg/ml of anti-CD62P antibody (Abcam) and washed in Tris-buffered saline (Dako Corporation). For antigen-antibody visualization the Ultra Vision LP Values Detection System (Lab Vision) together with DAB (3,30-diaminobenzidine) (DAKO corporation) were used. Finally, sections were washed and counterstained with Mayer’s haematoxylin.

### Enzyme-linked immunosorbent assay (ELISA)

Platelets from healthy controls, septic patients and septic thrombocytopenic patients were prepared as described above and the concentration of MMP-2 and MMP-9 measured in those samples using the Human ELISA Genie MMP-2 and MMP-9 kits (Reagent Genie Ltd, Dublin, Ireland) following the manufacturer instructions. The total protein concentration used for the assay was adjusted for all samples (100μg for plasma; 25 μg for lysate and 2.3 μg for homogenates).

### Statistical analysis

Patient data is reported as means or medians as appropriate. Categorical data was compared with Chi-squared tests. For all other results comparison of two groups was with independent samples t-tests or Mann-Whitney U tests. The Shapiro-Wilk test was used to test for normality. One-way analysis of variance (ANOVA) or Kruskal-Wallis (KW) tests were used for comparison of 3 groups, as appropriate, for data that was normally or non-normally distributed. Tukey’s honestly significant difference (HSD) is reported for multiple comparisons post ANOVA. For KW tests if a significant result was found pairwise comparison was performed using Dunn’s procedure with a Bonferroni correction for multiple comparisons and the adjusted p-values are reported. Comparison of 2 paired groups was with paired T-tests. If the data were not normally distributed the Wilcoxon Signed Rank test for related samples was used. Comparison of more than 2 paired groups used repeated measures ANOVA. If the assumption of sphericity was violated the Greenhouse-Geisser correction was applied. All analysis was performed with SPSS v20 software from IBM. A p value of less than 0.05 was considered statistically significant.

## Results

### Patient characteristics

A total of 26 septic patients were recruited, 31% developed thrombocytopenia either when admitted to the ICU or within 72 hours of admission (Table 2). The most common site of infection was intra-abdominal and the second was lung. Nineteen patients (73%) had a community-acquired infection and 7 a hospital-acquired or healthcare-associated infection. The most commonly identified organisms were gram-positive bacteria (30.8%) followed by gram-negative organisms (26.9%). Healthy controls were mostly male (60%) and had mean age of 40.2 years.

**Table 2:** Patient Demographics and Clinical characteristics.

	All patients	Non-thrombocytopenic	Thrombocytopenic	P-value for difference
**Number**	26	18	8	
**Age**	61.3	61	61.9	.873
**% Male**	58%	55.5%	62.5%	1.0
**APACHE II score**	27.6	28	26.7	.552
**SOFA score**	12.6	10.3	11.75	.125
**Survival to ICU discharge**	22/26 (84.6%)	17/18 (94.4%)	5/8 (62.5%)	.072
**Survival to hospital discharge**	18/26 (69.2%)	13/18 (72.2%)	5/8 (62.5%)	.667
**Median platelet count at admission (per μl)**	264,000	309,000	173,000	.944
**Median platelet count at sampling (per μl)**	236,500	267,000	84,000	.000*
**Median white cell count at sampling (per μl)**	13,400	15,000	10,350	.338

### Flow cytometry of Platelet MMP-2/-9, TLR-4 and P-selectin expression

**Fig 1:**
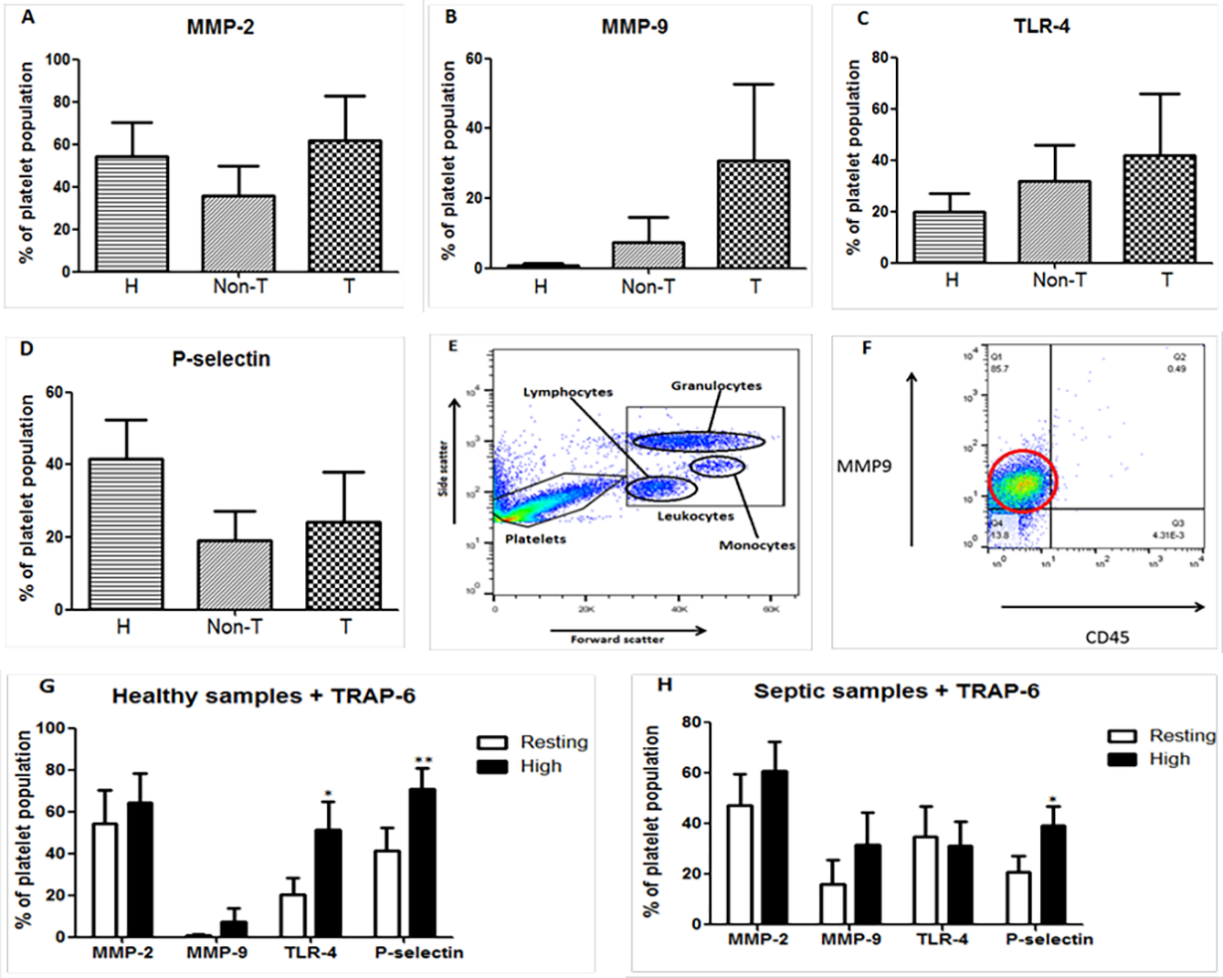
Flow cytometry (FC) analysis of whole blood samples from healthy controls (H), non-thrombocytopenic septic (Non-T) and septic thrombocytopenic (T) donors. **A–D**, Non-significant differences in the platelet surface expression of MMP-2, -9, TLR-4 and P-selectin between the 3 groups represented as the mean plus the standard error of the mean. **E**, Gating of the platelet population based on characteristic side and forward scatter. **F**, Representative graph from FC analysis in a thrombocytopenic septic patient demonstrating the presence of MMP-9 on platelets. The signal intensity of the MMP-9 antibody is highlighted by the red circle. In this case 85.7% of the gated platelet population is positive for the presence of MMP-9. **G**, Response of healthy platelets to agonist TRAP-6 with increased expression of TLR-4 and P-selectin. **H**, Response of platelets from septic patients to TRAP-6 with increased expression only in P-selectin. Data are mean ± SEM. n = 8 healthy controls, n = 5–9 non-thrombocytopenic donors, n = 3–5 thrombocytopenic donors. *p < 0.05 and **p < 0.01.

Whole blood samples obtained from patients and healthy subjects were first examined by FC in the absence of stimulation for platelet surface expression of MMP-2/-9, TLR-4 and P-selectin. There were no significant differences found in the platelet surface expression of these 4 proteins between healthy controls and septic donors in any of the analyzed subroups (Fig 1A–1F). Of note, MMP-9 was detected in small amounts in 2 out of 6 healthy control samples. With the addition of TRAP-6, MMP-9 was detected in a further 2 samples. In septic patients MMP-9 was detected in 6 out of the 8 patient samples. If platelet MMP-9 levels in healthy controls are independently compared to thrombocytopenic donors there is a significant increase noted (p = .048).

The platelet agonist TRAP-6 (25μM) was used to analyse the response of MMP-2, -9, TLR-4 and P-selectin to platelet activation (Fig 1G and 1H). MMP-2 did not increase with the addition of TRAP-6 in neither the healthy nor the septic groups. Similarly, no significant differences in MMP-9 were demonstrated in healthy controls or septic donors. TLR-4 expression was significantly upregulated in healthy controls with the addition of TRAP-6 (p = 0.028) but not in septic patients. Platelet P-selectin increased in both healthy controls (p = 0.002) and septic patients (p = 0.031).

### Flow cytometry of PLA formation

**Fig 2:**
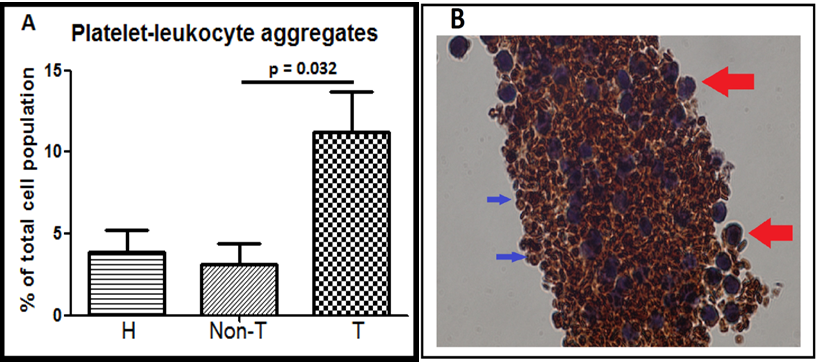
PLA formation in sepsis. **A**, Increased PLA formation in the thrombocytopenic (T) group (n = 4) compared to the non-thrombocytopenic (Non-T) group (n = 9); healthy (H) controls (n = 8). **B**, Immunohistochemical staining of a large PLA from a septic thrombocytopenic donor using a 10x objective lens magnification (100x). In the enhanced picture the large red arrows point to leukocytes and the smaller blue arrows point to platelets. Leukocytes are larger, nucleated and stained violet with haematoxylin. Platelets are smaller, stained with anti-CD62P and are rust in colour.

As described before, the population of PLAs were examined in whole blood by gating the CD45+/CD62P+ population [34]. When examined as 2 groups—healthy controls and septic patients–no differences were found. However when examined as 3 groups–healthy controls, septic non-thrombocytopenic patients and septic thrombocytopenic patients—significantly more PLAs had developed in the thrombocytopenic patients (p = .038). In a pairwise comparison, only a difference between the septic non-thrombocytopenic and septic thrombocytopenic group (p = .032) was found (Fig 2A). An example of a large PLA taken from a septic thrombocytopenic donor is shown in Fig 2B. In addition, all septic patients were examined according to their survival outcome and the development of PLAs. Although there was a trend towards increased formation of PLAs in non-survivors (mean 8% versus 3.5% in survivors) this was not statistically significant (p = .053).

### Examination of activity of gelatinases in sepsis using a 3 compartment model

**Fig 3:**
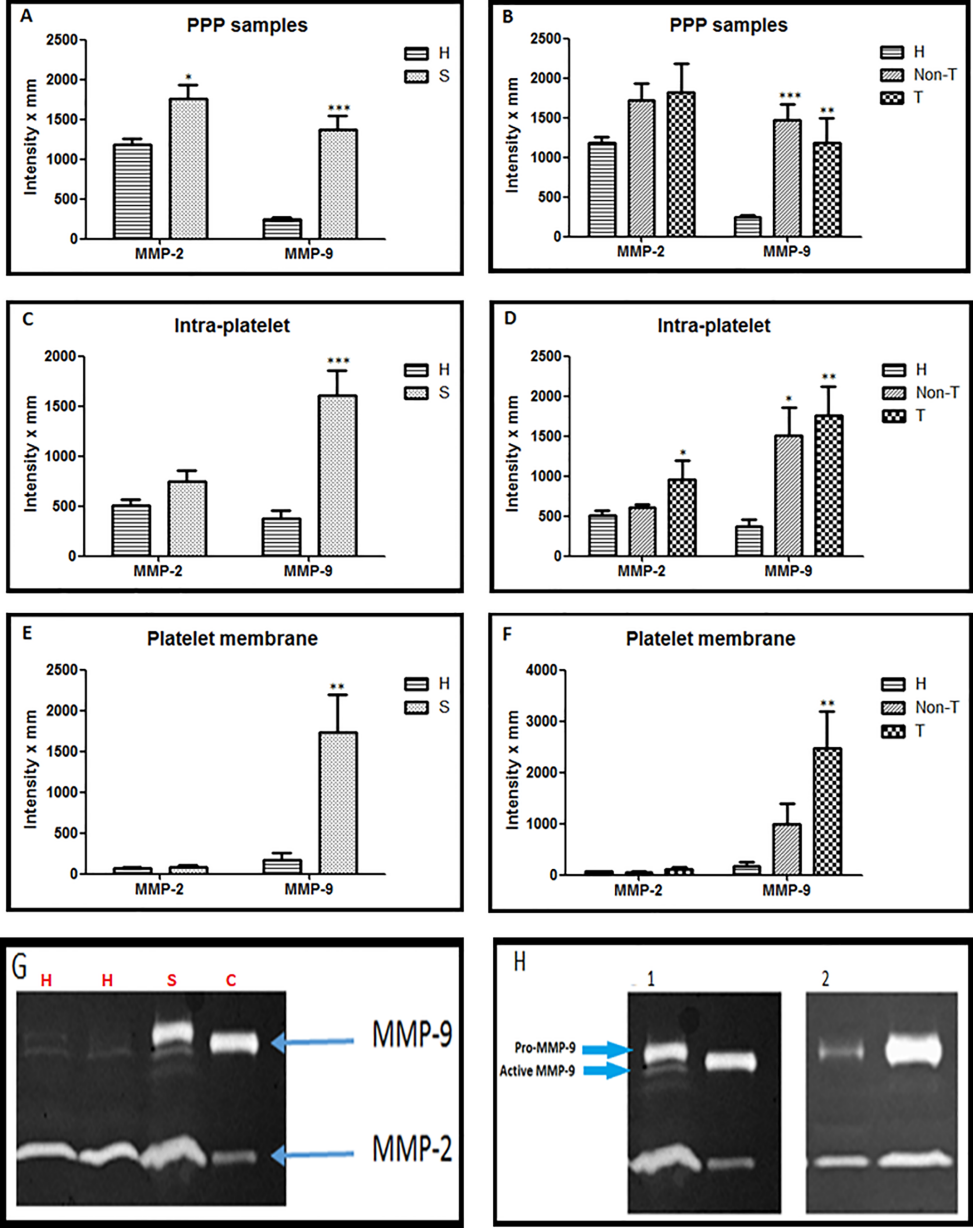
Examination of the activity of MMP-2 and -9 by gelatin zymography in septic shock as a 3 compartment model. Results are shown firstly as both healthy controls (H) and septic patients (S) and then with the septic patients categorised as non-thrombocytopenic (Non-T) and thrombocytopenic (T) patients. **A and B**, PPP samples at protein concentration (100μg/lane)–H (n = 20), Non-T (n = 13) and T (n = 7). **C and D**, Intraplatelet or lysate samples at protein concentration 30μg/lane–H (n = 10), Non-T (n = 7) and T (n = 5). **E and F**, Homogenate or plasma membrane samples at maximal protein concentration of 7.9 μg/lane–H (n = 9), Non-T (n = 5) and T (n = 5). **G**, Representative gel zymography showing both healthy controls (lane 1 and 2) and septic donors (lane 3); control = lane 4 (HT1080 media). **H**, Zymography gels. **1** shows PPP sample on the left lane and positive control on the right lane. On the left lane, a small band can be seen directly under the upper line. This small line corresponds to the active MMP-9 (82 kDa). This line is not visible in **2**, where lysate samples of septic patients were evaluated. Data are mean ± SEM. Results are compared to healthy controls *p < 0.05, **p < 0.01 and ***p < 0.001.

**Fig 4:**
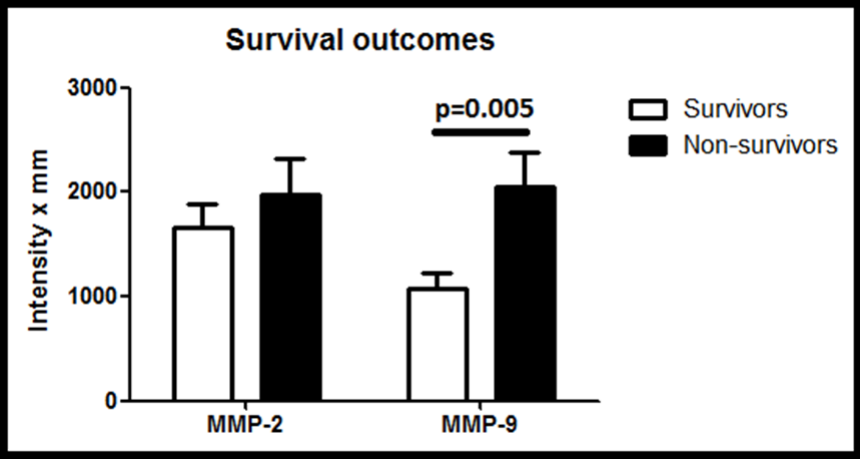
Survival outcomes. Plasma levels of MMP-9 were significantly higher in non-survivors (n = 6) compared to survivors (n = 14) (p = 0.005). No association was found between MMP-2 levels in PPP and mortality (p = 0.477).

MMP-2 activity was significantly higher in the plasma of septic patients versus healthy controls (Fig 3A). Levels of MMP-2 were similar in non-thrombocytopenic and thrombocytopenic patients (Fig 3B). MMP-9 activity in plasma was higher in septic patients compared to controls with no differences between the non-thrombocytopenic and thrombocytopenic patients (Fig 3B). Although intra-platelet MMP-2 activity was not modified when healthy controls and septic donors were compared, when examined as 3 groups it was significantly higher in the thrombocytopenic group (Fig 3C and 3D). Intra-platelet MMP-9 activity was higher in septic patients than controls, with similar activity in non-thrombocytopenic and thrombocytopenic patients (Fig 3C and 3D). Platelet membrane samples showed low activity of MMP-2 in both healthy and septic groups (Fig 3E and 3F). However, there was a significantly higher activity of MMP-9 found in the platelet membranes of septic patients compared to healthy controls with no differences between the non-thrombocytopenic and thrombocytopenic patients (Fig 3E and 3F). Plasma levels of MMP-2 and -9 in septic patients were compared based upon their survival and significantly higher MMP-9 levels were found in non-survivors (Fig 4).

To confirm our findings a series of ELISA experiments were performed using a small group of donors–healthy controls (n = 3), non-thrombocytopenic septic donors (n = 3) and thrombocytopenic septic donors (n = 3). Regarding MMP-2, as per the GZ results there was no significant difference in the intraplatelet and platelet membrane levels of MMP-2 between healthy controls and septic donors. An increase in plasma levels of MMP-2 in septic shock was not found using this technique. However, the presence of MMP-9 in platelets was confirmed with significant differences in the plasma (p = .016) and intraplatelet (p = .029) levels of MMP-9 in septic patients as compared to healthy controls. Contrary to the GZ findings platelet membrane expression of MMP-9 was not elevated in septic patients as compared to healthy controls. No significant difference was found between the non-thrombocytopenic and thrombocytopenic donors in MMP-2/-9 values in plasma, platelet membrane or intraplatelet compartment when examined with ELISA.

## Discussion

Given the significance of the development of thrombocytopenia in sepsis, approximately doubling the expected mortality, should the mechanism underlying SAT be clarified, it is likely that this would represent an appropriate target for the treatment of sepsis. The expression and activity of MMP-2 and MMP-9 was examined in patients with septic shock by FC and using a 3 compartment model (plasma, intraplatelet and platelet membrane) by gelatin zymography. Interestingly, both MMP-2 (in contrast to prior studies [35–37]) and MMP-9 were found to be increased in the plasma of septic patients. Importantly, higher levels of MMP-9, but not MMP-2, were found in plasma of patients who did not survive sepsis.

The presence of MMP-9 on platelets has been confirmed in this study under the conditions tested. In contrast to previous studies [38], we have detected platelet surface expression of MMP-9 in unstimulated whole blood samples by FC in 2 out of 6 healthy controls and 6 out of 8 septic donors. Sheu et al have previously shown intraplatelet expression of MMP-9 using immunogold labelling and electron microscopy [18]. Additionally we have demonstrated the presence of MMP-9 in platelets using zymography and ELISA techniques. It has been demonstrated that MMP-9 secretion from leukocytes significantly increases during inflammation and sepsis. Our findings suggest that platelet expression of MMP-9 is also upregulated in sepsis. In three studies where MMP-9 was not expressed by platelets, small groups of healthy controls were studied [17, 19, 39]. These differences in study populations may also account for some of the conflicting data from the literature. It was notable that when platelets were examined for the response to the platelet agonist TRAP-6, no significant increased level of MMP-9 was found. It may be that platelet expression of the gelatinases MMP-2 and -9 are independent of the PAR-1/PAR-4 platelet activation pathway [40].

Studies in larger cohorts have previously demonstrated increased platelet TLR-4 expression in patients suffering from sepsis [41]. Although platelet TLR-4 was not found to be upregulated in septic patients in this study, there appeared to be a trend of increased expression especially in the thrombocytopenic group. Two previous studies using genetically modified mice have demonstrated that TLR-4 deficiency attenuates lipopolysaccharide induced thrombocytopenia [21, 22]. The proposal that sepsis-associated thrombocytopenia is mediated via stimulation of TLR-4 receptors in platelets could not be corroborated in our study and it may be due to differences between humans and mice in the modulation of TLR expression [21]. We found that the addition of TRAP-6 increased TLR-4 expression on platelets in healthy controls but not in septic patients, possibly because TLR-4 expression had already been upregulated in this population. This finding is interesting as increased TLR-4 platelet expression in response to a platelet agonist has not been reported previously.

P-selectin expression was not found to be upregulated in septic patients. It has become increasingly recognised that PLAs are a better indicator of platelet activation *in vivo* than P-selectin platelet surface expression [42, 43]. We found an increased proportion of circulating PLAs in thrombocytopenic patients suggesting that consumption of a proportion of the circulating platelet population into PLAs could contribute to sepsis-associated thrombocytopenia. Interestingly, there is evidence that the release of MMPs such as MMP-2 and MMP-9 promotes generation of PLAs [34]. The role of PLAs in the pathophysiology of sepsis is unclear. Some authors have concluded that PLA formation is likely a significant pathological event contributing to microthrombi formation and the dysregulation of the microvasculature that is pathognomonic of sepsis [26, 44].

The main limitation of this study may be the small samples sizes involved as a result of the strict exclusion criteria employed. However, in order to capture clinically relevant findings in investigations we believe it is a better approach to apply stricter recruitment criteria despite the attendant problems with recruitment.

## Conclusions

Based on our results we believe that the consumption of platelets into platelet-leukocyte aggregates may contribute to a decline in platelet numbers in septic shock and therefore to the development of sepsis-associated thrombocytopenia. MMP-9 is found in platelets and may be an inducible enzyme in this setting.

## Supporting information

S1 File Data from FC and zymography experiments. (XLSX)
